# Supplementary material for: PROSTest, a Novel Liquid Biopsy Molecular Assay, Accurately Guides Prostate Cancer Biopsy Decision-Making in Men with Elevated PSA Irrespective of DRE Findings
Source: Cancers (Basel). 2025 Dec 6;17(24):3908. doi: 10.3390/cancers17243908 (PMC12730410; doi:10.3390/cancers17243908)
Supplement: Supplementary file 1 [file cancers-17-03908-s001.zip › cancers-3977071-supplementary.pdf]

SUPPLEMENTARY INFORMATION:

PROSTest, A Novel Liquid Biopsy Molecular Assay, Accurately Guides Prostate Cancer Biopsy Decision-Making in Men with Elevated PSA irrespective of DRE findings

Craig G. Rogers [1], Srinivas V. Koduru [2]*, Anthony Gulati [3] and Abdel B. Halim [2]

[1]**Vattikuti Urology Institute, Henry Ford Health, Detroit, Michigan, USA;** crogers2@hfhs.org
[2]**Wren Laboratories, Branford, Connecticut, USA.** skoduru@wrenlaboratories.com, ahalim@wrenlaboratories.com
[3]**Bennett Cancer Center, Stamford, Connecticut, USA;** agulati@stamhealth.org
***Correspondence:** skoduru@wrenlaboratories.com; **Tel.: +1(475)221-8056**

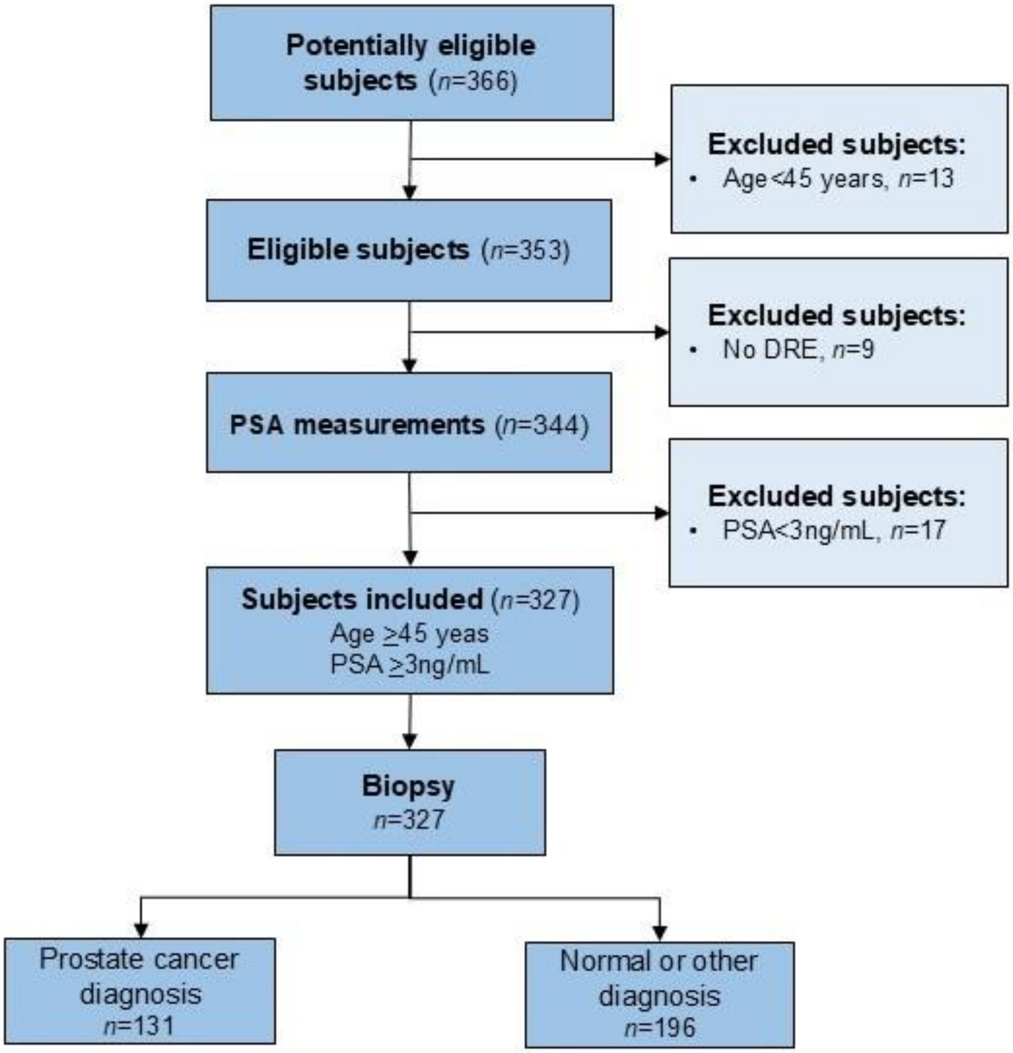

Supplementary Figure S1. Overview of the study and outcomes

3-10ng/mL

	DRE-positive cohort				DRE-negative cohort			
	Black		White		Black		White	
	PCa+	PCa-	PCa+	PCa-	PCa+	PCa-	PCa+	PCa-
PROSTest+ve	2	4	26	1	0	9	38	2
PROSTest-ve	0	37	0	14	0	38	2	42
PPV	33.3%		96.3%		N/A		95.0%	
NPV	100.0%		100.0%		N/A		95.5%	
Accuracy	90.7%		97.6%		N/A		95.2%	

>10ng/mL

	Black		White		Black		White	
	PCa+	PCa-	PCa+	PCa-	PCa+	PCa-	PCa+	PCa-
PROSTest+ve	0	2	28	0	0	6	31	0
PROSTest-ve	0	7	1	7	0	21	2	7
PPV	N/A		100.0%		N/A		100.0%	
NPV	N/A		87.5%		N/A		77.8%	
Accuracy	N/A		97.2%		N/A		95.0%	

Supplementary Figure S2A. Tables (2x2) and diagnostics metrics (PPV: positive predictive value, NPV: negative predictive value and accuracy) for ethnicity in DRE positive and negative individuals separated by PSA cohort (Cohort 1: 3-10ng/mL and Cohort 2: >10ng/ml).
N/A reflects not analyzed (insufficient data for analysis).

3-10ng/mL	DRE-positive cohort				DRE-negative cohort			
	Family Hx		No Family Hx		Family Hx		No Family Hx	
	PCa+	PCa-	PCa+	PCa-	PCa+	PCa-	PCa+	PCa-
PROSTest+ve	1	0	27	4	6	4	32	7
PROSTest-ve	0	11	1	40	0	10	2	70
PPV	100.0%		87.1%		100.0%		82.1%	
NPV	100.0%		97.6%		100.0%		97.2%	
Accuracy	100.0%		93.1%		100.0%		91.9%	

>10ng/mL	Family Hx		No Family Hx		Family Hx		No Family Hx	
	PCa+	PCa-	PCa+	PCa-	PCa+	PCa-	PCa+	PCa-
PROSTest+ve	1	0	27	2	2	1	29	5
PROSTest-ve	0	3	1	11	0	6	2	22
PPV	N/A		93.1%		66.7%		85.3%	
NPV	N/A		91.7%		100.0%		91.7%	
Accuracy	N/A		92.7%		88.9%		87.9%	

Supplementary Figure S2B. Tables (2x2) and diagnostics metrics (PPV: positive predictive value, NPV: negative predictive value and accuracy) for family history of prostate cancer in DRE positive and negative individuals separated by PSA cohort (Cohort 1: 3-10ng/mL and Cohort 2: >10ng/mL).

Hx = history; N/A reflects not analyzed (insufficient data for analysis).

3-10ng/mL	DRE-positive cohort				DRE-negative cohort			
	≤75 years		>75 years		≤75 years		>75 years	
	PCa+	PCa-	PCa+	PCa-	PCa+	PCa-	PCa+	PCa-
PROSTest+ve	25	3	3	1	34	11	4	0
PROSTest-ve	1	51	0	0	2	75	0	5
PPV	89.3%		N/A		75.6%		100.0%	
NPV	98.1%		N/A		97.4%		100.0%	
Accuracy	95.0%		N/A		89.3%		100.0%	

>10ng/mL	≤75 years		>75 years		≤75 years		>75 years	
	PCa+	PCa-	PCa+	PCa-	PCa+	PCa-	PCa+	PCa-
PROSTest+ve	23	2	5	0	28	6	3	0
PROSTest-ve	1	12	0	2	2	28	0	0
PPV	92.0%		100.0%		82.4%		N/A	
NPV	92.3%		100.0%		93.3%		N/A	
Accuracy	92.1%		100.0%		87.5%		N/A	

Supplementary Figure S2C. Tables (2x2) and diagnostics metrics (PPV: positive predictive value, NPV: negative predictive value and accuracy) for age (cut-off of 75 years(in DRE positive and negative individuals separated by PSA cohort (Cohort 1: 3-10ng/mL and Cohort 2: >10ng/ml). N/A reflects not analyzed (insufficient data for analysis).

Supplemental Table S1: PROSTest Marker Genes (*n*=27)

Symbol	Name	NCBI Chromosome Location	UniGene ID	RefSeq	GO Biological Process*
AAMP	Angio-associated migratory cell protein	Chr.2: 218264129 - 218270209	Hs.83347	NM_001087.4	0001525 (angiogenesis) 0010595 (endothelial cell migration)
AR	Androgen receptor (Δ exon boundary – AR-2)	Chr.X: 67544032 - 67730619	Hs.76704	NM_000044.3	0000122 (transcription) 0000165 (MAPK cascade)
CHTOP	Chromatin Target Of PRMT1	Chr.1: 153633982 - 153646306	Hs.611057	NM_001206612.1	0001701 (embryonic development) 0006338 (chromatin remodeling)
EDC4	Enhancer Of MRNA Decapping 4	Chr.16: 67873023 - 67884514	Hs.75682	NM_014329.4	0031087 (mRNA decapping)
FXYD7	FXYD Domain Containing Ion Transport Regulator	Chr.19: 35143250 - 35154302	Hs.134729	NM_022006.1	0006811 (monoatomic ion transport)
FYCO1	FYVE And Coiled-Coil Domain Containing 1	Chr.3: 45917899 - 45995824	Hs.200227	NM_024513.3	0072385 (vesicle transport) 1901098 (autophagasome maturation)
HNRNPU	Heterogeneous Nuclear Ribonucleoprotein U	Chr.1: 244842123 - 244864720	Hs.106212	NM_004501.3	0000122 (transcription) 0000381 (spliceosome)
HPN	Hepsin	Chr.19: 35040506 - 35066573	Hs.182385	NM_002151.2	0006508 (proteolysis) 0008360 (regulation cell shape)
KRT23	Keratin 23	Chr.17: 40922696 - 40937643	Hs.9029	NM_001282433.1	0030855 (epithelial cell differentiation) 0045109 (filament organization)
MAN2B2	Mannosidase Alpha Class 2B Member 2	Chr.4: 6575174 - 6622403	Hs.188464	NM_001292038.1	0005975 (carbohydrate metabolism)
MAX	MYC Associated Factor X	Chr.14: 65006101 - 65102695	Hs.285354	NM_001320415.1	0000122, 0006355 (transcription)
MRPS25	Mitochondrial Ribosomal Protein S25	Chr.3: 15042251 - 15065337	Hs.657764	NM_022497.4	0032543 (mitochondrial translation)
NDUFS2	NADH: Ubiquinone Oxidoreductase Core Subunit S2	Chr.1: 161197377 - 161214395	Hs.173611	NM_001166159.1	0006120 (mitochondrial electron transport)
PPRC1	Peroxisome Proliferator-Activated Receptor Gamma, Coactivator-Related 1	Chr.10: 102132994 - 102150333	Hs.533551	NM_001288727.1	0006351 (transcription)
RAD23A	RAD23 Homolog A, Nucleotide Excision Repair Protein	Chr.19: 12945814 - 12953643	Hs.643267	NM_001270362.1	0006281 (DNA repair)

REPIN1	Replication Initiator 1	Chr.7: 150368228 - 150374044	Hs.647086	NM_013400.3	0006260 (DNA replication)
SDR39U1	Short Chain Dehydrogenase/Reductase Family 39U Member 1	Chr.14: 24439766 - 24442905	Hs.643552	NM_020195.2	None
SETBP1	SET Binding Protein 1	Chr.18: 44680173 - 45068510	Hs.435458	NM_001130110.1	0006338 (chromatin remodeling)
SLC14A1	Solute Carrier Family 14 Member 1 (Kidd Blood Group)	Chr.18: 45724123 - 45752520	Hs.101307	NM_001128588.3	0006833 (water transport)
SLC18A2	Solute Carrier Family 18 Member A2	Chr.10: 117241073 - 117279430	Hs.596992	NM_003054.4	0006836 (neuro-transmitter transport)
SMC4	Structural Maintenance of Chromosomes 4	Chr.3: 160399304 - 160434962	Hs.58992	NM_001002800.2	0000070 (chromatid segregation)
SPARC	Secreted Protein Acidic and Cysteine Rich	Chr.5: 151661096 - 151687054	Hs.111779	NM_001309443.1	0001937 (endothelial cell proliferation)
SQLE	Squalene Epoxidase	Chr.8: 124998478 - 125022283	Hs.71465	NM_003129.3	0006629 (lipid metabolism)
STRIP1/ FAM40A	Striatin Interacting Protein 1	Chr.1: 110031577 - 110054641	Hs.584996	NM_001270768.1	0003674 (molecular function)
STX12	Syntaxin 12	Chr.1: 27773183 - 27824452	Hs.523855	NM_177424.2	0000045 (autophagasome assembly)
UNC45A	Unc-45 Myosin Chaperone A	Chr.15: 90929980 - 90954093	Hs.389461	NM_001039675.1	0007517 (muscle organ development)
XPC	XPC Complex Subunit, DNA Damage Recognition and Repair Factor	Chr.3: 14145147 - 14178672	Hs.475538	NM_004628.4	0006281 (DNA repair)

*GO Biological Role: http://geneontology.org/ [27,28]

Supplementary Table S2: Study Cohort demographics (*n*=327)

	**PCa cohort**	**Non-PCa cohort**	***p*-value***
Number	131	196	N/A
Age	67 [51-86]	65 [46-88]	0.041
Ethnicity: W:B (% W)	129:2 (98.5%)	72:124** (36.7%)	*<0.0001†*
Family History of PCa	10 (7.6%)	35 (17.9%)	*0.0086†*
DRE +ve	58 (44.3%)	71 (36.2%)	0.145†
PSA (ng/ml)	9.98 [3.2-101]	6.0 [3-174]	<0.0001
PROSTest (+ve, %) ‡	125 (95.4%)	23 (11.7%)	<0.0001†
PROSTest scores	88.1 [8.2-99.2]	11.8 [1.2-85.5]	<0.0001

Data are median [range] or as a % ( )
**2-tailed Mann-Whitney U-test*
***2 mixed ethnicities are included in the Black group.*
†2-way Chi^2 test
‡ This includes the % of subjects with a positive (≥50%) PROSTest score.
N/A = not applicable, DRE = digital rectal examination, W = White, B = Black
